# Printed Electrodes Based on Vanadium Dioxide and Gold Nanoparticles for Asymmetric Supercapacitors

**DOI:** 10.3390/nano13182567

**Published:** 2023-09-16

**Authors:** Bashaer A. Minyawi, Mohammad Vaseem, Nuha A. Alhebshi, Amal M. Al-Amri, Atif Shamim

**Affiliations:** 1 Department of Physics, Faculty of Science, King Abdulaziz University, Jeddah 21589, Saudi Arabia; 2 Integrated Microwave Packaging Antennas and Circuit Technology (IMPACT) Lab, Computer, Electrical and Mathematical Sciences and Engineering (CEMSE) Division, King Abdullah University of Science and Technology (KAUST), Thuwal 23955-6900, Saudi Arabia; 3 Department of Physics, College of Science and Arts, King Abdulaziz University, Rabigh 21911, Saudi Arabia

**Keywords:** ink, screen printing, supercapacitor, thin films, vanadium dioxide, nanoparticles

## Abstract

Printed energy storage components attracted attention for being incorporated into bendable electronics. In this research, a homogeneous and stable ink based on vanadium dioxide (VO_2_) is hydrothermally synthesized with a non-toxic solvent. The structural and morphological properties of the synthesized material are determined to be well-crystalline monoclinic-phase nanoparticles. The charge storage mechanisms and evaluations are specified for VO_2_ electrodes, gold (Au) electrodes, and VO_2_/Au electrodes using cyclic voltammetry, galvanostatic charge–discharge, and electrochemical impedance spectroscopy. The VO_2_ electrode shows an electrical double layer and a redox reaction in the positive and negative voltage ranges with a slightly higher areal capacitance of 9 mF cm^−2^. The VO_2_/Au electrode exhibits an areal capacitance of 16 mF cm^−2^, which is double that of the VO_2_ electrode. Due to the excellent electrical conductivity of gold, the areal capacitance 18 mF cm^−2^ of the Au electrode is the highest among them. Based on that, Au positive electrodes and VO_2_ negative electrodes are used to build an asymmetric supercapacitor. The device delivers an areal energy density of 0.45 μWh cm^−2^ at an areal power density of 70 μW cm^−2^ at 1.4 V in the aqueous electrolyte of potassium hydroxide. We provide a promising electrode candidate for cost-effective, lightweight, environmentally friendly printed supercapacitors.

## 1. Introduction

Energy and power demands in our society are rising quickly and steadily each year because of the growing population, global development, and generally improved standards of living [1]. The primary source of energy is fossil fuels, which have negative environmental effects, such as pollution of water and air. It is crucial to move toward renewable energy, including wind and solar resources, which can provide a long-term solution for sustainable development. However, solar and wind are intermittent energy sources [2]. It is therefore valuable to expand advanced energy conversion/storage techniques. Through the concept of energy storage, these renewable resources can be made to be reliable and steady energy sources. In addition, electrical energy storage systems are serious elements for electric vehicles and portable electronics in everyday life [3]. Therefore, energy storage technologies much attention is paid to developing their performance. 

Generally, the most recognized energy storage devices are batteries such as aqueous Zn-ion batteries [4,5], Li-ion batteries [6], lithium–selenium batteries [7], and ammonium-ion batteries [8], conventional capacitors, and electrochemical capacitors, which can also be named supercapacitors [9,10]. Supercapacitors are split into two groups according to their charge storage mechanism and research and development trends. The first type is the electric double-layer capacitor (EDLC), and the second is the pseudocapacitor (PC). When the two mechanisms of charge storage become functionalized simultaneously, a new class of supercapacitors is created, which is called the hybrid supercapacitor (HC). The electrochemical responses of the electrode materials, the electrolyte, and the voltage range all affect supercapacitor performance. The most crucial element at the core of the technique is the electrode. The selection of electrodes and their fabrication are crucial in determining the performance of a supercapacitor. Surface area, electrical conductivity, thermal stability, electrode wettability, corrosion resistance, and electrolyte solution permeability are all important factors in the electrochemical performances of electrode materials [11]. They should also be low-cost and environmentally sustainable as well. Commonly, five categories of electrode materials are most used: carbon derivatives [12], transition metal composites [13], metal–organic frameworks [14], MXene compounds [15], and conducting polymers [16].

The rapid advancement of technology requires the development of smart electronic materials with the ability to tune their properties on demand. In particular, metal–insulator transition (MIT) materials hold considerable potential in their simple and reversible tunability between the insulator and metal when applied with external stimuli [17]. Many MIT materials are available, such as V_2_O_3_, RNiO_3_, Fe_3_O_4_, Ti_2_O_3_, and VO_2_ [18]. At a particular temperature, their electrical conductivity drastically changes, which known as the transition temperature. Among them, vanadium-based compounds, including vanadium oxides [19] and vanadium nitrides [20], have the ability to tune their electrical and optical properties, are low-priced, have safe chemistry [21], elevated specific capacity, and multiple oxidation states [22]. Vanadium dioxide (VO_2_) received increased attention due to it is possessing an insulating state at room temperature and a metallic state at a low transition temperature (340 K). When the temperature is lower, VO_2_ exhibits a monoclinic structure (M-phase). However, when the temperature is above that, the VO_2_ takes on a rutile structure (R-phase) [23,24]. The vanadium-derived electrode is known as one of the most effective active materials for supercapacitors due to its excellent electrochemical performance [25]. Vanadium dioxide (VO_2_) is safe and compatible with various electrolytes. Synthesis methods and performance of VO_2_-based supercapacitors are compared in Table 1. 

The mixed-phase VO_2_ electrodes performed worse than the pure-phase electrode in the Na_2_SO_4_ electrolyte, which had 33 mF cm^−2^ areal capacitance at 10 mV s^−1^, and a capacitance retention of 93.7% after 5000 cycles [26]. Asymmetric supercapacitors consisting of different combined electrodes basically have higher operating voltages than symmetric supercapacitors. The VO_2_(M) electrode pairs well with alkaline electrolytes such as KOH. With 49.28 mAh g^−1^-specific discharge capacity and 663 F g^−1^-specific capacitance at a scan rate of 5 mV s^−1^, it shows outstanding performance [28]. In a separate illustration, a symmetric supercapacitor based on VO_2_ was used with 1 M LiClO_4_ in a propylene carbonate organic electrolyte, which demonstrated 46 Wh kg^−1^ at 1.4 kW kg^−1^ of energy and power densities [29]. These models depend on conventional techniques for making electrodes, such as mixing the precipitated VO_2_ with conducting materials and binders such as carbon and carboxymethyl cellulose, and manually casting the mixture onto sheets. The generated electrodes are typically bulky, hence the difficulty of their use with on-chip integrated circuits since these approaches need expensive equipment and intricate procedures.

Innovative printed supercapacitors drew interest for being integrated as energy storage elements into the Internet of Things (IoT) and smart fabrics due to their low weight, excellent power density, and safety. One of the main advantages of printed devices is the fast production and the low potential cost [32]. In general, there are two types of manufacturing methods for electronic devices: additive manufacturing processes and conventional subtractive manufacturing processes. Subtractive manufacturing is fundamentally based on removing material. First, the material is deposited on a surface, followed by etching the pattern; then, the excess material is removed usually by lithographic patterning, lift-off, and etching [33]. Conventional technologies are not desirable for preparing electronic devices and are difficult to apply as fabrication strategies because it is time consuming, has a very high total cost, and produces much waste material during the removal process, which is an environmental concern in this manufacturing. On the contrary, additive manufacturing processes can be completed in a single step of deposition of patterned materials on a specific area on flexible substrates. As a result, both fabrication time and material utilization are reduced. Many methods of additive manufacturing are used as material jetting and extrusion, binder jetting, powder bed fusion, and vat photopolymerization [34]. Among them, screen printing, 3D printing, and inkjet printing are printing-based methods that provide a quick and accurate method to deposit the materials on a designed region of a variety of sheets, with much less material waste and fewer processing steps compared to conventional manufacturing [35]. This procedure gives sustainable fabrication in many applications. Considering that, not only can supercapacitors [35] be printed in large quantities for flexible electronics, but also switches of radio-frequency electronics [36], photovoltaics [37], transistors [38], optoelectronics [39], and transistors [38].

Screen printing is an additive manufacturing technique in the printed electronics process whereby ink is squeezed and applied to a surface using a stencil screen [40]. Screen printing is a simple and flexible process that may be utilized with many functional inks and substrates [35]. A number of inks are created, including graphene ink [41,42], polymer nanowires ink [43], silver nanoparticles ink [44], silver organo-complex ink [45], and ink with VO_2_ nanoparticles [17]. However, the ink’s concentration and viscosity have a significant impact and need for optimization. Different two-dimensional material-based inks were screen printed onto flexible micro-supercapacitors [43]. For example, 2D titanium carbide MXene-based micro-supercapacitors showed an amazing areal capacitance of 158 mF cm^−2^ and a superb energy density of 1.64 Wh cm^−2^ [46]. The creation of high-quality ink for incredibly thin printed layers is a primary obstacle affecting the widespread use of printed electrodes.

To the best of our knowledge, few articles were published to investigate screen-printed vanadium oxide electrodes for supercapacitors. In one article [27], a hydrothermal method was used to synthesize monoclinic VO_2_(B) nanorods. Then, acetylene black and polyvinylidene fluoride (PVDF) was merged in N-methyl pyrrolidone (NMP) to mix with the nanorods. Through a mesh of 100 mesh counts, an around 0.15 mm-thickness film was printed on Ni foam and performed 64 and 99 F g^−1^-specific capacitances, respectively, at 1 and 0.5 A g^−1^. Nevertheless, VO_2_(M) is more stable than VO_2_(B) [47]. Moreover, both PVDF and NMP are dangerous and costly. It is recommended to replace them by other binders in water-based solvents [48]. Another article is reported by our group [30] where a screen printer was used to print VO_2_(M) microparticle-based ink that contains nontoxic ethyl cellulose (EC) on Kapton substrate. It is worth mentioning that the single layer-printed symmetric supercapacitor revealed an areal energy of 0.2 μWh cm^−2^ at 17.5 μW cm^−2^ in a 3 M KOH aqueous electrolyte within 1.4 V. 

Herein, our aim is to improve the electrochemical performance of screen-printed supercapacitors based on VO_2_ ink. This is achieved using a toxic-free preparation of VO_2_(M) nanoparticles instead of the above mentioned examples. The performance is further improved by hybridizing with gold nanoparticles. The results are investigated in both half-cell and full-cell configurations. The full-cell asymmetric supercapacitor delivers an areal energy density of 0.45 μWh cm^−2^ at an areal power density of 70 μW cm^−2^ at 1.4 V. This research direction could foster applications of lightweight, sustainable, and environmentally friendly energy storage components for flexible electronics chips and smart textiles.

## 2. Materials and Methods

### 2.1. Synthesis of VO_2_ Nanoparticles

The materials used in the synthesis are vanadium (IV), oxide sulfate hydrate (VOSO_4_·xH_2_O, 97% anhydrous, Sigma-Aldrich, Burlington, MA, USA), urea (NH_2_CONH_2_, Fisher Scientific, Waltham, MA, USA), hydrazine hydrate (N_2_H_4_, 50–60%, reagent grade, Sigma-Aldrich), and ethanol (C_2_H_5_OH, absolute, VWR Chemicals, Radnor, PA, USA).

The nanoparticles preparation of VO_2_ was processed by a hydrothermal method [17]. Briefly, 0.1 M vanadium (iv) oxide sulfate hydrate was mixed with 150 mL of deionized (DI) water containing 1.8 g of urea. The resulting solution became a clear-blue mixture, and then 0.9 mL hydrazine hydrate (10% hydrazine hydrate solution in water) was inserted drop by drop, with stirring, and put in an autoclave at 260 °C. Then, the blue-black precipitate was collected by centrifuging, cleaning, and annealing in a vacuum furnace at 70 °C for 1 h. Then, the resultant powder was annealed in a vacuum furnace at 300 °C for 3 h, becoming pure VO_2_ (M) nanoparticles. 

### 2.2. VO_2_ Ink Preparation

First, a mixture solution was formulated of terpineol and ethanol to keep suitable ink viscosity and surface tension. Interestingly, ethyl cellulose (EC) binder was added as a dispersive agent and rheological modifier. The mixture solution consists of 74 weight% of terpineol, 18.5 weight% of ethyl cellulose, and 7.5 weight% of ethanol. Finally, this prepared solution was agitated with the obtained pure VO_2_ particles at a 3:5 weight ratio to produce a homogenous, stable, and screen-printable VO_2_ ink.

### 2.3. Electrodes Printing and Testing

**Figure 1:**
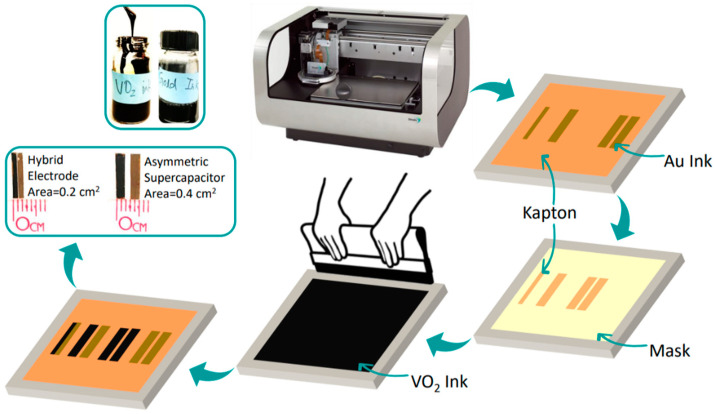
Scheme of electrodes printing. Photograph of the inkjet printer and steps of screen printing technique, the inset photographs show ink bottles of VO_2_ and Au nanoparticles, the VO_2_/Au half-cell hybrid electrode, and the VO_2_//Au full-cell asymmetric supercapacitor.

The electrodes fabrication steps are illustrated in Figure 1. Starting with Kapton, substrate was cleaned with ethanol and distilled water and then dried by nitrogen gas. In the second step, a polymeric mask was cut in a parallel rectangles design. To obtain a hybrid electrode of VO_2_ and gold together (VO_2_/Au), commercial conductive nanogold ink from C-INK CO., LTD., Soja City, Japan (DryCure Au-J, 10 wt% solid Au with viscosity 10 cps) was printed by an inkjet printer (Dimatix DMP-2831 from Fujifilm Incorporation, Valhalla, NY, USA) on Kapton substrate. Because of its low viscosity, it is not suitable for printing with the manual screen printer. In simplified terms, the printing process is carried out in a DOD printer in a piezoelectric inkjet process with a nozzle diameter of 16 µm; the drop volume was 10 pL, and an electric field distorts the nozzle based on the shape of the digital design specified by the user. Then, pressure is applied to force the ink through the nozzle onto the substrate. The uniform and continuous ejection of droplets was achieved while applying a firing voltage of 16–18 V at a 5 kHz printer velocity. The cartridge temperature was set to room temperature. The two subsequent layers were printed with an average film thickness of less than 1 µm using drop spacing of 20 µm. After printing the two layers, curing was conducted for 1 h at 120 °C.

Then, on Kapton substrate and using a mesh of 325 counts, the VO_2_ ink was manually printed. After that, the painted sheet was sintered at 120 °C for 1 h. It should be noted that the temperature of 120 °C is used to evaporate the solvents (terpineol and ethanol) from the printed films. The ethyl cellulose is not decomposed at this temperature; rather, it retains and helps to maintain the flexibility of printed films. For the pure VO_2_ electrode, the previous process is preceded without the Au printing.

There are two types of cell arrangements for electrochemical measurements to determine supercapacitor electrode performance and characterize the electrode material. The first type is a half-cell, which is also called a three-electrode cell, and the other type is a full-cell, also called a two-electrode cell. In the three-electrode system, a working electrode of our sample, a counter electrode of a platinum plate, and a reference electrode of silver/silver chloride (Ag/AgCl) were used [49]. The thickness of our printed VO_2_ is ~10–15 µm.

Two types of electrodes were tested in half-cells, which are 0.2 cm^2^ electrodes of vanadium dioxide nanoparticles (VO_2_), and 0.2 cm^2^ electrode hybrid electrodes of VO_2_ and gold (VO_2_/Au), each printed on 0.1 cm^2^. For full-cells, the VO_2_ electrode and Au electrode were separately cut off and dipped together into the electrolyte solution as an asymmetric supercapacitor VO_2_//Au. The electrochemical performances of all half-cells and full-cells were evaluated at room temperature by cyclic voltammetry (CV), galvanostatic charge–discharge (GCD), and electrochemical impedance spectroscopy (EIS). The electrochemical workstation (CHI 660D model, CH Instruments Incorporation, Austin, TX, USA) was used.

The areal capacitance (C_A_) in Farad per square centimeters (A cm^−2^) can be specified by the geometric electrode area (A), the voltage window (∆V), the current (I), and the discharge time (∆t) with the following equation:

(1) $$C_{A}=\frac{I\Delta t}{A\Delta V}$$

The worthiest merits determining the performance of energy devices are energy and power densities, which are also directly relevant to the end applications [50]. The energy (E) in joles (J), and the power (P) in watts (W) stored in a capacitor are, respectively, determined by equations [51]:

(2) $$E=\frac{1}{2}CV^{2}$$

(3) $$P=\frac{E}{\Delta t}$$

### 2.4. Materials Characterization

Structural characterization techniques are necessary for nanostructured electrode materials. Most of these material characterization techniques can be categorized as either microscopy or spectroscopy. To determine the crystal phase of our sample, an X-ray diffraction system (XRD, Bruker D8 ADVANCE, Billerica, MA, USA) equipped with a CuK source (0.15406 nm) was used. Two models of scanning electron microscopes (SEM, Merlin Model, ZEISS, Oberkochen, Baden-Württemberg, Germany, and Magellan 400 Model, FEI Company, Hillsboro, OR, USA) were utilized at 2.0 kV to explore the shape of the VO_2_ particles. Both morphology and crystallinity are also investigated by a transmission electron microscope (TEM, FEI Titan G2 80–300 kV Model, FEI Company, Hillsboro, OR, USA) equipped with a 2 k × 2 k charge-coupled device (CCD) camera. The pore volume distribution, in addition to the specific surface area, was calculated by Brunauer–Emmet–Teller (BET) and Barret–Joyner–Halender (BJH) methods, respectively. These calculations were derived from the N_2_ adsorption–desorption isotherm at −195.75 °C, using an accelerated surface area and porosimetry system (High-Resolution Surface Area and Porosimetry-3Flex 3500, Norcross, GA, USA). Moreover, the thickness of the prepared VO_2_ was calculated using the Dektak XT stylus surface profiler (Bruker Corporation, Billerica, MA, USA).

## 3. Results and Discussion

### 3.1. Structural and Morphological Properties

**Figure 2:**
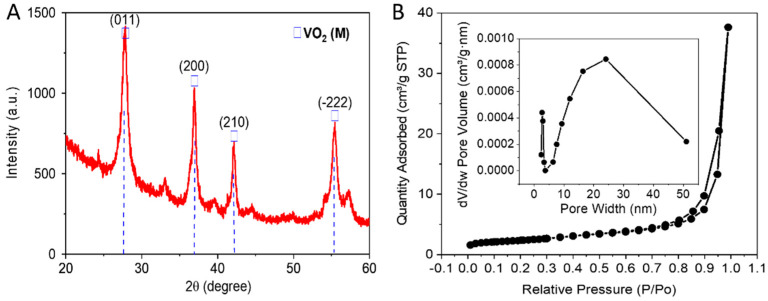
Structural characterization of VO_2_ nanoparticles. (**A**) XRD spectrum of VO_2_ nanoparticles. (**B**) N_2_ adsorption–desorption isotherm with Barret–Joyner–Halender pore–volume destruction curve.

After the preparation of VO_2_ nanoparticles, XRD analysis is conducted to examine the crystalline phase. The XRD spectrum explained in Figure 2A shows that well-crystallized VO_2_ (M) phases are obtained after synthesizing with a 6 h reaction time and annealing at 300 °C for 3 h in a vacuum. XRD peaks are located at 27.84°, 37.01°, 42.19°, and 55.56°, which are assigned to the (011), (200), (210), and (−222) crystal atomic planes, respectively, according to the Joint Committee on Powder Diffraction Standards (JCPDS) No. 72-0514 [52]. Further characterization is crucial. 

For VO_2_ nanoparticles, the N_2_ adsorption–desorption isotherm is established in Figure 2B. A slight hysteresis loop signifies a mesoporous form. The BET surface area of 8.38 m^2^ g^−1^ is calculated from the isotherm. Huang et al. [53] reported a higher BET surface area of 29.1 m^2^ g^−1^ for VO_2_(B) nanorods synthesized hydrothermally for 15 h, which is a longer reaction time than our synthesis method of VO_2_(M) nanoparticles. Moreover, VO_2_(M) is more stable than VO_2_(B) [47]. Furthermore, the inset of Figure 2B shows the BJH-derived pore volume distribution of the sample. For the pores of less than 40.3 nm-width, the total pore volume is 0.0286 cm^3^ g^−1^ at a $p/p_{0}$ of 0.95. Such features of good surface area and mesopores can enhance the electrode–electrolyte interface, which is required in supercapacitors materials.

**Figure 3:**
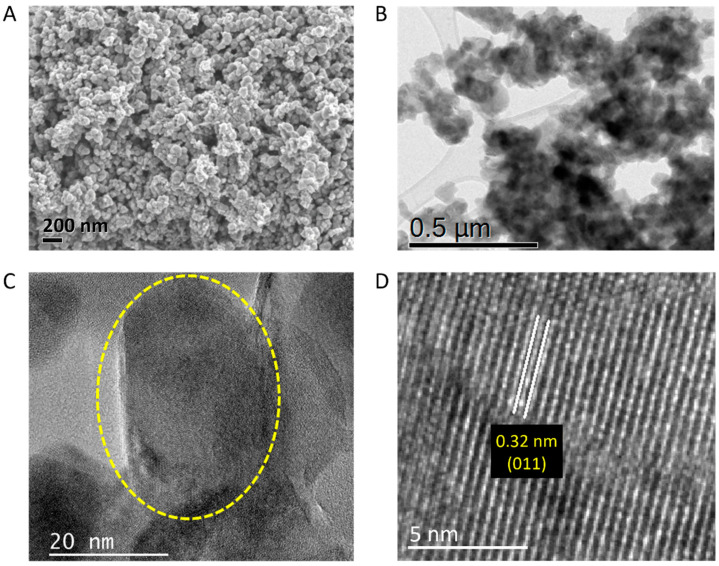
Morphological characterization of VO_2_ nanoparticles. (**A**) SEM image. (**B**–**D**) TEM images where (**D**) image is enlarged from the yellow circle region of (**C**) image.

Furthermore, mostly spherical and aggregated nanoparticles with an average particle size smaller than 100 nm are imaged by SEM in Figure 3A for the annealed VO_2_. Such nanoparticles are promising because they could possess different properties than the bulk size of the same materials [54]. The particle measurements were checked by their TEM images. Low-resolution and high-resolution TEM images are captured in Figure 3B–D. The morphology of nearly spherical nanoparticles with an average aggregate size of about 40 nm is indicated by the circle in Figure 3C. In addition, Figure 3D shows that the interplanar distance was measured as 0.32 nm, which is related to the (011) crystal plane. This is in agreement with the XRD spectra and d-spacing value. Similar TEM results were published in a scientific paper by our team [17]. 

### 3.2. Electrochemical Performance

#### 3.2.1. Evaluation of Half-Cell Electrodes 

Figure S1 in the supporting information document illustrates the electrochemical storage characteristics of the VO_2_ nanoparticles electrode in 3 M KOH. Indicating that the charge storage mechanism is primarily caused by the quick creation of an electric double layer (EDL), the cyclic voltammetry curves in Figure S1A demonstrate a predicted quasi-rectangular curve at fast scan rates between −0.15 V and 0.55 V voltage window. At low scan rates, mild faradic redox processes, as well as oxygen evolution, are responsible for the large peaks in the CV in Figure S1B. In line with that, Figure S1C,D shows that the GCD curves have a modest departure from the triangle shape, which can be attributed to a significant EDL form and moderate redox reactions.

Nanogold (Au) is one of the common metal electrode materials, with ideal conductivity that can enhance the electrochemical performance of our supercapacitors. The electrochemical storage performance of Au electrodes is depicted in Figure S2 in the Supplementary material, demonstrating the effect of applying different voltage scan rates on the CV current. At higher scan rates, CVs exhibit nearly rectangular behavior as shown in Figure S2A. Both reduction (cathodic) and oxidation (anodic) current peaks in Figure S2B are visible at low scan rates, indicating the fast redox reactions of Au. In agreement with that, the charge–discharge processes are confirmed in Figure S2C,D. 

**Figure 4:**
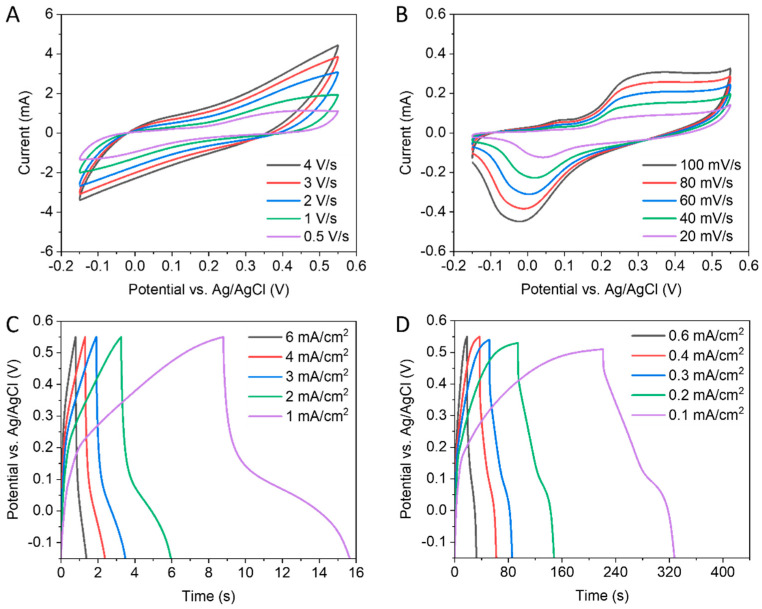
Electrochemical performance of VO_2_/Au hybrid electrodes from −0.15 to 0.55 V: (**A**,**B**) CV curves. (**C**,**D**) GCD curves.

Figure 4 points out the electrochemical storage performance of the hybrid electrode VO_2_/Au. The CV curves shown in Figure 4A,B indicate that both mechanisms of EDL and redox reaction would increase the capacitance. The GCD behavior of the VO_2_/Au at areal current within the range from 6 to 0.1 mA cm^−2^ is plotted in Figure 4C,D. The electrical conductivity of Au in the hybrid electrode is anticipated to improve the electrochemical charging–discharging.

**Figure 5:**
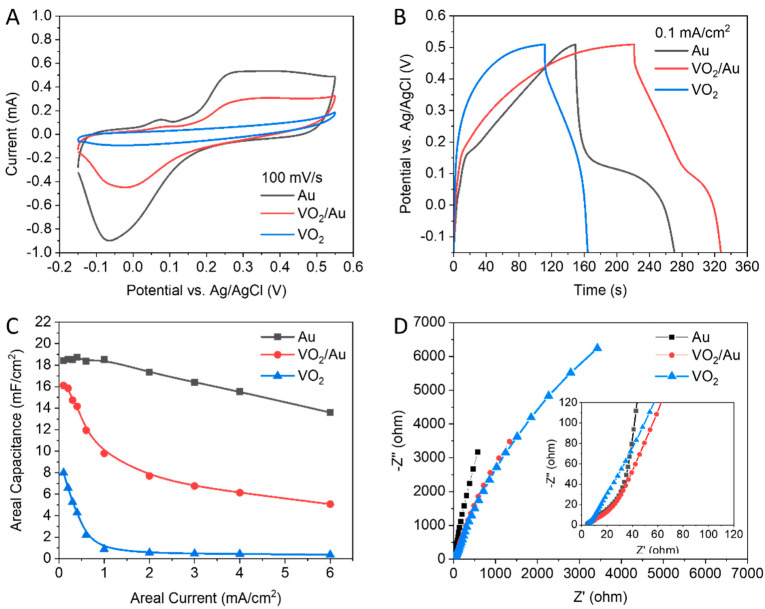
Comparisons of hybrid VO_2_/Au electrodes and their individual components from −0.15 to 0.55 V. (**A**) CV curves. (**B**) GCD curves. (**C**) Areal capacitance functions. (**D**) Nyquist plot of EIS with an enlarged scale.

To compare the electrochemical performance of hybrid VO_2_/Au electrodes and their components electrodes, CV characteristics were performed, as shown in Figure 5A. The Au electrode resulted in a significantly broader CV curve than that of VO_2_/Au electrode at 100 mV/s of the same scan rate. On the other hand, the hybrid electrode is better than VO_2_ itself. It is well recognized that Au has a superior electrical conductivity. Nevertheless, the conductivity of VO_2_/Au is sufficient to achieve excellent electrochemical performance. Furthermore, in Figure 5B, the comparison between our electrodes at 0.1 mA cm^−2^ shows that the GCD curve of the Au electrode has the longest discharging time and hence higher areal capacitances. Furthermore, from the charging/discharging areal current, we determined the areal capacitance (C_A_) using Equation (1) from data of GCD curves. Consequently, the areal capacitances of the Au and VO_2_/Au electrodes are higher than the results of the VO_2_ electrode. At 0.1 mA cm^−2^, it is calculated that the maximum areal capacitance of 18.393 mF cm^−2^ is attained for the Au electrode and becomes 16.106 mF cm^−2^ for a VO_2_/Au electrode, nearly twice the areal capacitance for the pure VO_2_ electrode, as shown in Figure 5C. In Figure 5D, the Nyquist plot of EIS with an enlarged scale at the inset is conducted in a 3 M KOH electrolyte. The ESR of the Au, VO_2_/Au, and VO_2_ electrodes are estimated to be ~4.36, 5, and 4.3 ohms, respectively. At a high-frequency range, the VO_2_/Au owns a similar performance to the Au, but at a low frequency, the VO_2_/Au is better than VO_2_.

**Figure 6:**
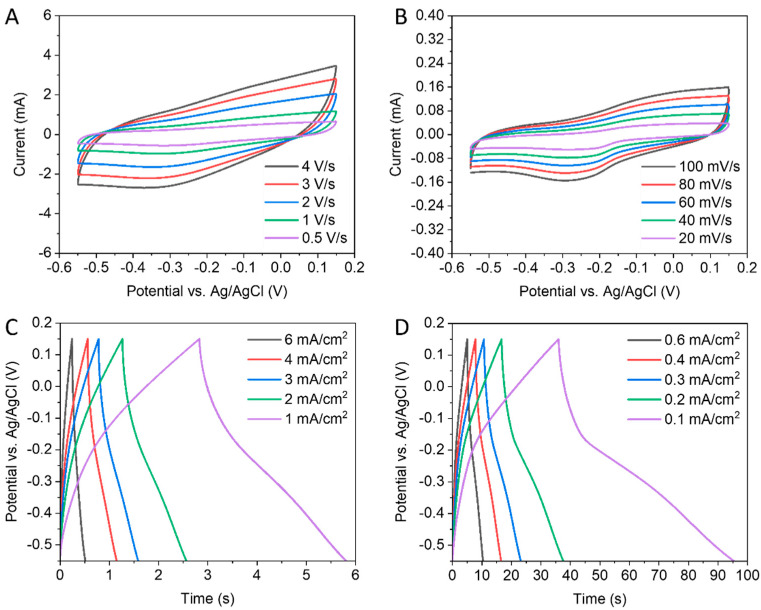
Electrochemical performance of VO_2_ electrode from −0.55 to 0.15 V. (**A**,**B**) CV curves. (**C**,**D**) GCD curves.

Before making the full-cell, it is suggested to examine the half-cell of the VO_2_ electrode in the negative potential window. In Figure 6A,B, the CV curves indicate that there are oxidation and reduction peaks. In addition, a good redox reversibility is implied by the small and stable potential displacement between the peaks. Consequently, the time of charging and discharging processes in Figure 6C,D suggests that the VO_2_ electrode works on the negative voltage side with better electrochemical performance than on the positive side abovementioned in Figure S1C,D. At 0.1 mA cm^−2^, the VO_2_ electrode shows a slightly higher 8.5 mF cm^−2^ areal capacitance than that of VO_2_ in the positive range (8.0 mF cm^−2^).

#### 3.2.2. Performance of Full-Cell Supercapacitors

**Figure 7:**
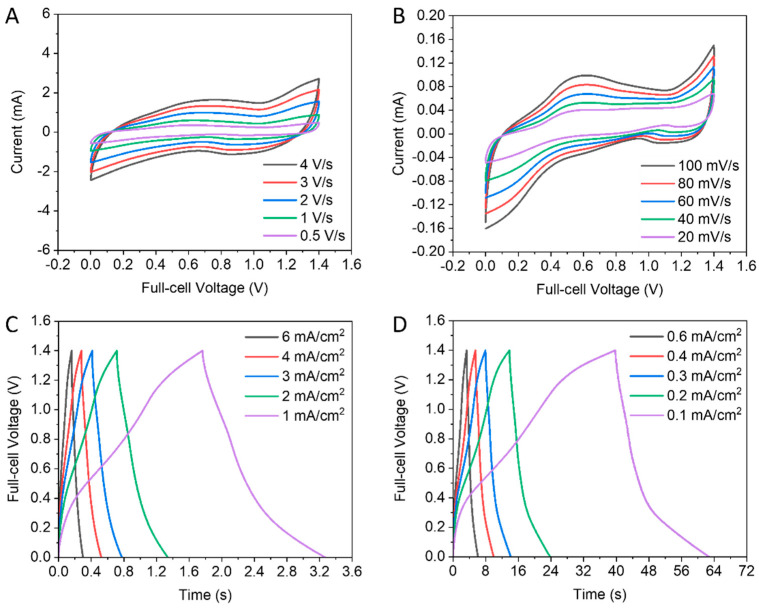
Electrochemical performance of VO_2_//Au asymmetric supercapacitor from 0 to 1.4 V. (**A**,**B**) CV curves. (**C**,**D**) GCD curves.

The electrochemical storage performance of our materials was subsequently assessed in full-cells based on the encouraging half-cell results. Asymmetric supercapacitors were constructed, with the VO_2_ electrode as the negative electrode due to its excellent electrochemical storage performances in the negative voltage range, as discussed in the previous section, and Au as the positive electrode. Figure 7 reveals the supercapacitor results of the VO_2_//Au full-cell. The CV curves demonstrated that during a broader potential range of 1.4 V, the electrical double layers and reversible redox reactions occurred. This is an advantage of the asymmetric design of supercapacitors. Our aqueous asymmetric supercapacitor has a broad stable voltage value caused by the deployment of distinct redox reactions in anode and cathode. Likewise, The GCD curves of the full-cell are demonstrated in Figure 7C,D at different areal currents. The full-cells of asymmetric supercapacitors are successfully charged and discharged at the same areal current values that used in half-cell testing.

**Figure 8:**
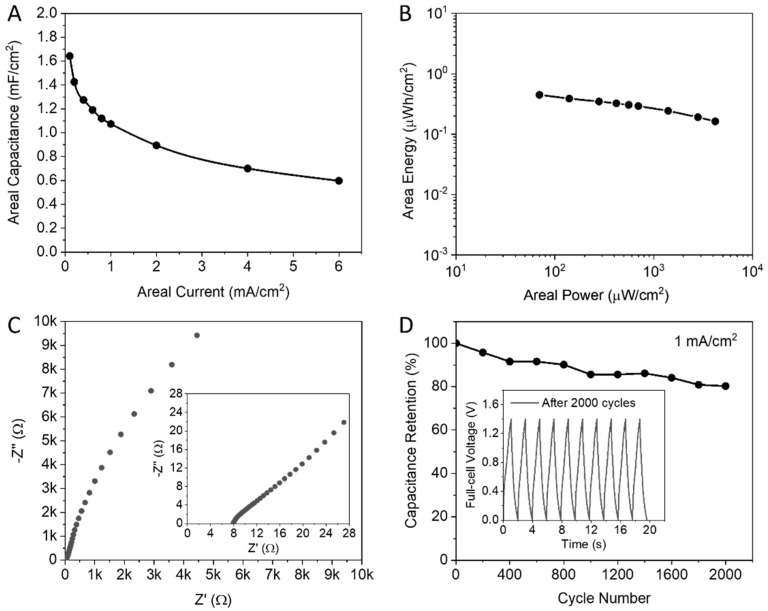
Electrochemical performance of VO_2_//Au asymmetric supercapacitor. (**A**) Areal capacitances. (**B**) Ragone plot. (**C**) Nyquist plots from EIS with enlarged scale in the inset. (**D**) Capacitance retention with GCD cycles in the inset.

We calculated the areal capacitance according to the GCD curves, 1.642 mF cm^−2^ was recorded at 0.1 mA cm^−2^, as pointed in Figure 8A. Additionally, the areal energy values of this supercapacitor are calculated from the areal capacitances and operating voltage (Equation (2)), then the areal power values are calculated using Equation (3) and plotted in Figure 8B. The device delivers areal energy of 0.447 μWh cm^−2^ at an areal power of 70 μW cm^−2^. Moreover, the full-cell has around 7.9 Ω of equivalent series resistances (Figure 8C). Figure 8D shows that 80% of the capacitance is retained after 2000 GCD cycles at 1 mA cm^−2^. In comparison, the area, energy, and power of this VO_2_//Au asymmetric supercapacitor are superior than that published for symmetric VO_2_ microparticles, which are 0.2 μWh cm^−2^ at 17.5 μW cm^−2^ [30]. A maximum areal energy of 0.68 μWh cm^−2^ at an areal power of 95 μW cm^−2^ is recorded by Velmurugan et al. [31] using a V_2_O_5_ symmetric supercapacitor with PVA-KOH. Nevertheless, their V_2_O_5_ samples were fabricated by physical vapor deposition with vacuum coating units, which is a complex and costly preparation method. The printing techniques used in our study are recommended for simple, time-saving, and cost-effective fabrication methods. 

## 4. Conclusions

VO_2_ nanoparticles were prepared in a solution followed by annealing. The XRD and SAED patterns correspond to a well-crystalline (M) phase of VO_2_ with an interplanar distance of 0.32 nm for (011) crystal planes. The SEM and TEM images confirm the nanoparticles morphology with a size range between 20 and 50 nm. The BET surface area of our nanoparticles is found to be 8.38 m^2^/g; such a large area is attributed to the presence of mesopores as calculated from the N_2_ adsorption–desorption isotherm. The VO_2_ electrode, Au electrode, and VO_2_/Au electrode were tested as half-cells in KOH electrolytes using CV, GCD, and EIS techniques. The VO_2_ electrode shows an electrical double layer in the positive voltage range and a redox reaction in the mostly negative range with a slightly higher 9 mF cm^−2^ areal capacitance at 0.1 mA cm^−2^. The VO_2_/Au electrode displays 16 mF cm^−2^, which is double that of the VO_2_ electrode in the mostly positive voltage window. Due to the excellent electrical conductivity of gold, the areal capacitance 18 mF cm^−2^ of the Au electrode is the highest among them. Based on that, the Au electrode and VO_2_ electrode are used to build an asymmetric supercapacitor. The device delivers 0.45 μWh cm^−2^ at 70 μW cm^−2^ at 1.4 V in an aqueous KOH electrolyte. Moreover, the full-cell has around 7.9 Ω of equivalent series resistances, and 80% of the capacitance is retained after 2000 GCD cycles. These electrochemical performance results and the fast preparation method provide a promising electrode candidate for lightweight, environmentally friendly, and practically printed supercapacitors.

## Figures and Tables

**Table 1:** Comparisons of VO_2_-based supercapacitor’s performance.

Electrode	Max. C_A_ at I_A_ or v	Max. E_A_ at P_A_	Reference
VO_2_ nanoporous	33 mF cm^−2^ at 10 mV s^−1^	1.5 mWh cm^−2^ at ~150 mW cm^−2^	Basu et al. [26]
VO_2_ nanorods	99 F g^−1^ at 1 A g^−^1	Not reported	Zhang et al. [27]
VO_2_ nanosheets	663 F g^−1^ at 5 mV s^−1^	Not reported	Ndiaye et al. [28]
VO_2_ nanosheet	405 F g^−1^ at 1 A g^−1^	46 Wh kg^−1^ at 1.4 kW kg^−1^	Rakhi et al. [29]
VO_2_ microparticles	0.2 mF cm^−2^ at 5 mA cm^−2^	0.2 μWh cm^−2^ at 17.5 μW cm^−2^	Alhebshi et al. [30]
V_2_O_5_ thin film	5 mF cm^−2^ at 0.125 mA cm^−2^	0.68 μWh cm^−2^ at 95 μW cm^−2^	Velmurugan et al. [31]

C_A_: Areal capacitance, I_A_: areal current, v: scan rate, E_A_: areal energy, and P_A_: areal power.

## Data Availability

Data sharing is not applicable to this article.

## Supplementary Materials

The following supporting information can be downloaded at: https://www.mdpi.com/article/10.3390/nano13182567/s1, Figure S1. Electrochemical Performance of VO_2_ Electrode from −0.15 to 0.55 V. (A,B) CV curves. (C,D) GCD curves. Figure S2. Electrochemical Performance of Au Electrode from −0.15 to 0.55 V: (A,B) CV curves. (C,D) GCD curves.
