# Supplementary material for: Printed Electrodes Based on Vanadium Dioxide and Gold Nanoparticles for Asymmetric Supercapacitors
Source: Nanomaterials (Basel). 2023 Sep 16;13(18):2567. doi: 10.3390/nano13182567 (PMC10535297; doi:10.3390/nano13182567)
Supplement: Supplementary file 1 [file nanomaterials-13-02567-s001.zip › nanomaterials-2588374-supplementary.pdf]

Supporting Information

Printed Electrodes Based on Vanadium Dioxide and Gold Nanoparticles for Asymmetric Supercapacitors

Bashaer A. Minyawi [1], Mohammad Vaseem [2], Nuha A. Alhebshi [1,*], Amal M. Al-Amri [3], and Atif Shamim [2]

[1] Department of Physics, Faculty of Science, King Abdulaziz University, Jeddah 21589, Saudi Arabia; bmakhdhurminyawi@stu.kau.edu.sa, nalhebshi@kau.edu.sa

[2] IMPACT Lab, Computer, Electrical and Mathematical Sciences and Engineering (CEMSE) Division, King Abdullah University of Science and Technology (KAUST), Thuwal 23955–6900, Kingdom of Saudi Arabia; mohammad.vaseem@kaust.edu.sa, atif.shamim@kaust.edu.sa

[3] Department of Physics, College of Science and Arts, King Abdulaziz University, Rabigh 21911, Saudi Arabia; amsalamri@kau.edu.sa

* Correspondence: nalhebshi@kau.edu.sa

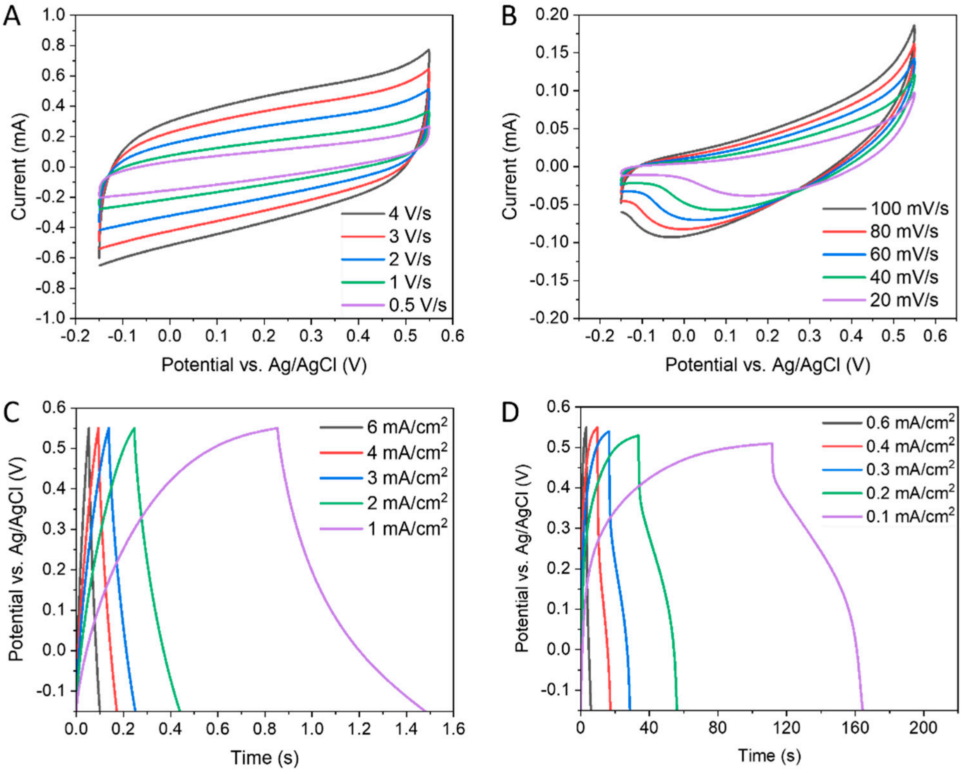

Figure S1. Electrochemical Performance of VO_2 Electrode from -0.15 to 0.55 V. (A, B) CV curves. (C, D) GCD curves.

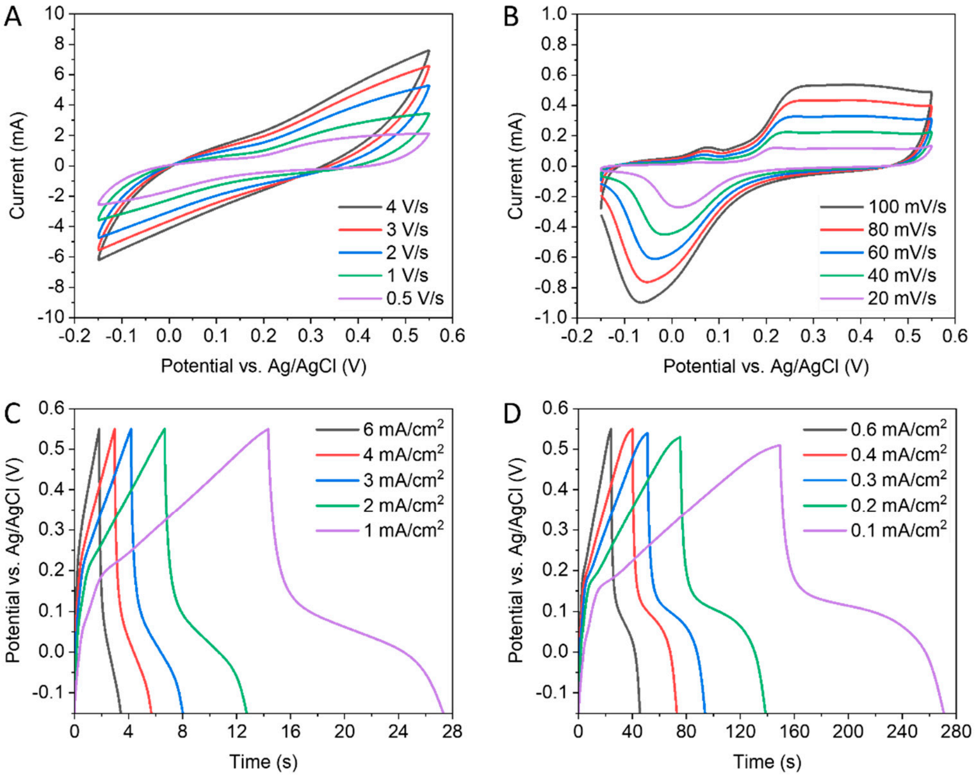

Figure S2. Electrochemical Performance of Au Electrode from -0.15 to 0.55 V: (A, B) CV curves. (C, D) GCD curves.
